# On the Growth of Scientific Knowledge: Yeast Biology as a Case Study

**DOI:** 10.1371/journal.pcbi.1000320

**Published:** 2009-03-20

**Authors:** Xionglei He, Jianzhi Zhang

**Affiliations:** 1 State Key Laboratory of Biocontrol, School of Life Sciences, Sun Yat-sen University, Guangzhou, China; 2 Department of Ecology and Evolutionary Biology, University of Michigan, Ann Arbor, Michigan, United States of America; University of Chicago, United States of America

## Abstract

The tempo and mode of human knowledge expansion is an enduring yet poorly understood topic. Through a temporal network analysis of three decades of discoveries of protein interactions and genetic interactions in baker's yeast, we show that the growth of scientific knowledge is exponential over time and that important subjects tend to be studied earlier. However, expansions of different domains of knowledge are highly heterogeneous and episodic such that the temporal turnover of knowledge hubs is much greater than expected by chance. Familiar subjects are preferentially studied over new subjects, leading to a reduced pace of innovation. While research is increasingly done in teams, the number of discoveries per researcher is greater in smaller teams. These findings reveal collective human behaviors in scientific research and help design better strategies in future knowledge exploration.

## Author Summary

It is of great interest to understand the patterns and mechanisms of scientific knowledge growth, but such studies have been hampered by the lack of ideal cases in which the structure of the knowledge is known, the knowledge is quantifiable, and the process of knowledge discovery is well understood and documented. The biological knowledge about a species is in part described by its protein interaction network and genetic interaction network. Here, we conduct a temporal meta-analysis of three decades of discoveries of protein interactions and genetic interactions in baker's yeast to reveal the tempo and mode of the growth of yeast biology. We show that the growth is exponential over time and that important subjects tend to be studied earlier. However, expansions of different domains of knowledge are highly heterogeneous and episodic such that the temporal turnover of knowledge hubs is much greater than that expected by chance. Familiar subjects are preferentially studied over new subjects, leading to a reduced pace of innovation. While research is increasingly done in teams, the number of discoveries per researcher is greater in smaller teams. These findings reveal collective human behaviors in scientific research and help design better strategies in future knowledge exploration.

## Introduction

Scientific knowledge refers to the body of facts and principles that are known in a given field. Modern civilization is built on the knowledge that humans have acquired about the world they live in, and the future of the human species and society critically depends on further accumulation of scientific knowledge. Patterns and mechanisms of human knowledge growth are jointly determined by the intrinsic structure of knowledge and human behaviors in knowledge exploration. Although such behaviors are of interest to many scientists including philosophers [1],[2], sociologists [3], anthropologists [4], economists [5], physicists [6], and psychologists [7], they are poorly studied, due primarily to the lack of ideal cases in which (i) the structure of the knowledge is known, (ii) the knowledge is quantifiable, and (iii) the process of knowledge discovery is well understood and documented.

As biologists, we notice that the above three requirements are all met for biological knowledge of the baker's yeast *Saccharomyces cerevisiae*. Knowledge can be described largely as relationships among a set of subjects. Over the past three decades, scientists have substantively deepened their understanding of yeast biology through the study of interactions among its ∼6000 genes [8]. By the end of 2007, over 73,000 yeast gene-gene interactions had been discovered and documented in ∼5,400 publications authored by 11,238 researchers (see Materials and Methods). Much of the structure of the knowledge about yeast biology can be described as a gene-gene interaction network, where the unit of knowledge is an interaction. Scientific publications record the approximate date of each relevant discovery, as well as the methodology used. As a case study, we here analyze the temporal growth of the known yeast gene-gene interactions to understand the tempo and mode of scientific knowledge expansion.

## Results

### Exponential Growth and Productivity of Individuals

Gene-gene interactions are separated into two types: genetic interactions (GIs) and protein-protein interactions (PPIs) [9]. Two genes are said to interact genetically if the effect of one gene on a trait is masked or enhanced by the other. Two genes are said to have a PPI if their protein products physically bind to each other stably or transiently. The data we considered contain 37,809 PPIs among 4,913 genes and 35,231 GIs among 3,743 genes, respectively (see Materials and Methods). Because of the difference in the nature of PPIs and GIs, we study the yeast PPI and GI networks separately.

**Figure 1:**
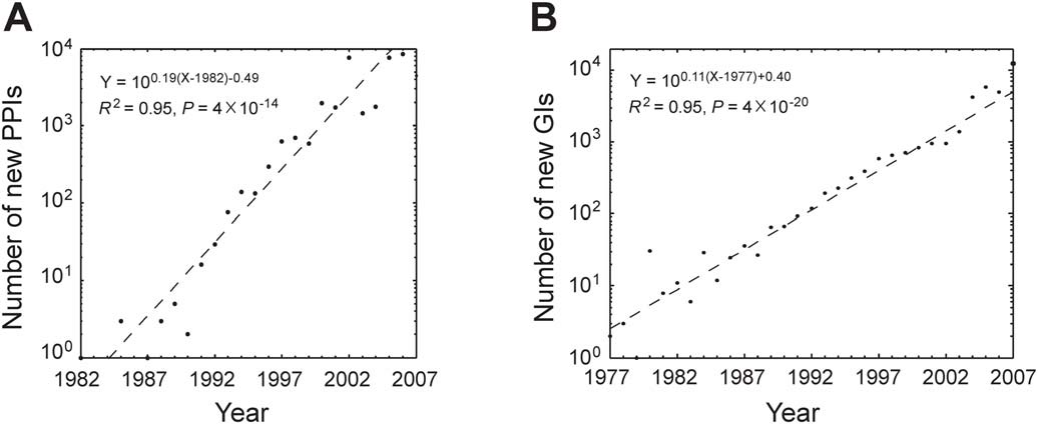
Numbers of new interactions discovered each year in the yeast (A) protein-protein interaction (PPI) network and (B) genetic interaction (GI) network. The data of 2007 are not considered in the fitting because we downloaded the yeast PPI and GI data from BioGRID in July 2007.

**Figure 2:**
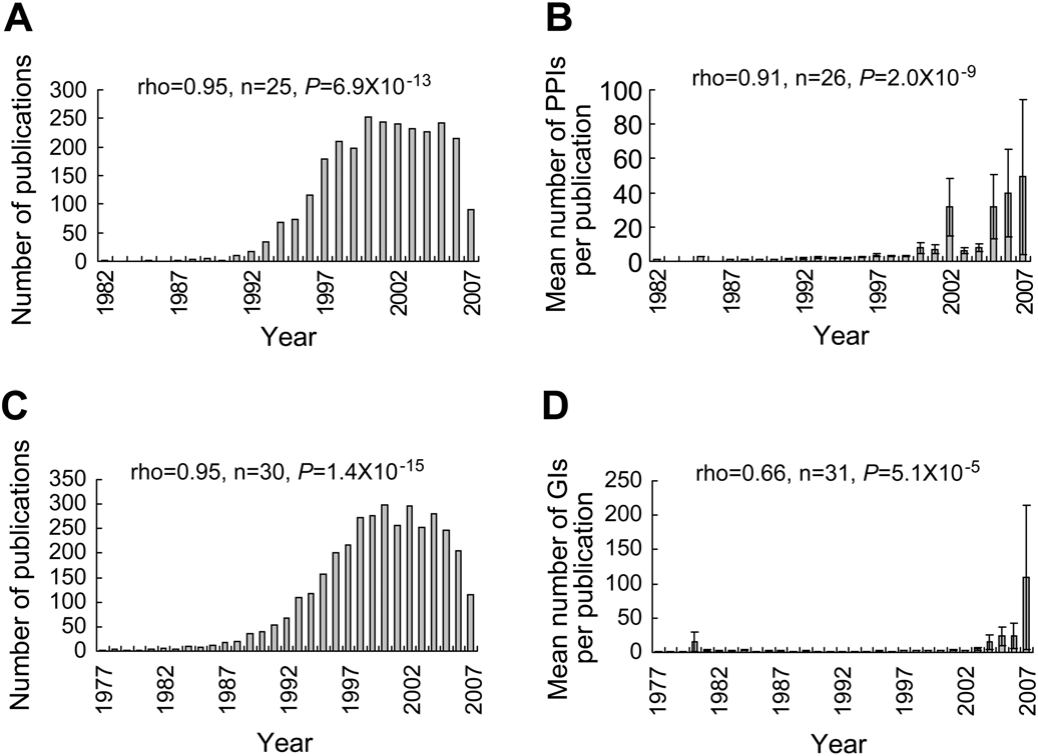
Increased numbers of studies and productivity per study over time. Error bars show one standard error of the mean. (A) Number of publications per year reporting PPIs increases over time. (B) Mean number of novel PPIs discovered per study increases over time. (C) Number of publications per year reporting GIs increases over time. (D) Mean number of novel GIs discovered per study increases over time. *P* is two-tailed *P*-value for the statistical significance of Spearman's rank correlation (ρ).

**Figure 3:**
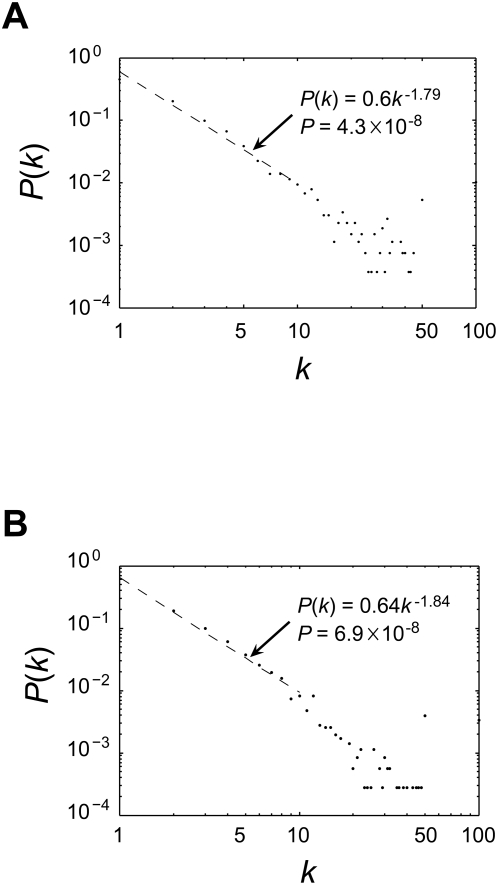
The power-law distribution of productivity per study for (A) PPIs and (B) GIs. The dotted line shows the fitting for *k*≤10, which includes ∼93% and ∼96% of considered publications for PPIs and GIs, respectively. Publications with *k* from 50 to 99 were lumped together and plotted at *k* = 50, and publications with *k*≥100 were lumped together and plotted at *k* = 100.

**Figure 4:**
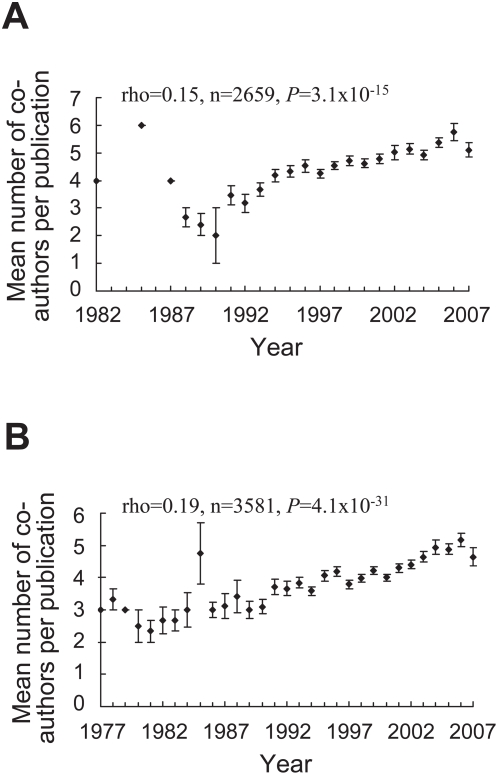
The number of co-authors per publication reporting (A) PPIs and (B) GIs increased over time. Error bars show one standard error of the mean. *P* is two-tailed *P*-value for the statistical significance of Spearman's rank correlation (ρ).

**Figure 5:**
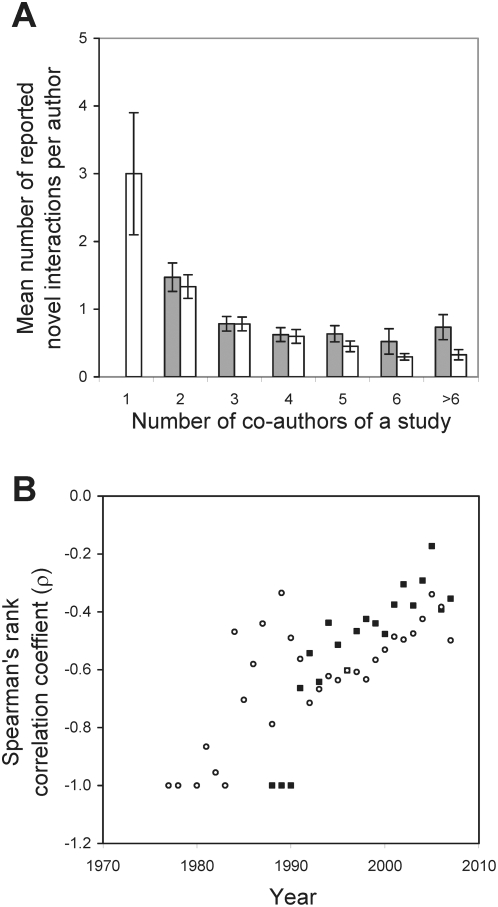
Mean number of novel PPIs (grey bars) or GIs (white bars) discovered per author in a study reduces as the number of co-authors of the study increases for the papers published in any given year. (A) Results from year-1998 are shown here as an example (*n* = 210 papers, Spearman's rank correlation ρ = −0.424, *P* = 1.4×10^−10^ for PPIs; *n* = 273 papers, ρ = −0.634, *P*<10^−15^ for GIs). Error bars show one standard error. (B) All years show a negative rank correlation (ρ) between the number of novel PPIs (black squares) or GIs (white circles) reported per author in a study and the number of co-authors of the study. Statistical significance of the correlations can be found in Table S2.

The PPI data were published from year-1982 to 2007, spanning 26 years, while the GI data were published from year-1977 to 2007, spanning 31 years (see Materials and Methods). The number of new interactions discovered per year increased approximately exponentially over time (Figure 1), and there is no apparent sign of slowing of this exponential growth at present. The exponential growth can be attributed to the increased number of studies per year and/or the enhanced productivity per study over time (Figure 2). *P*(*k*), the probability that a study discovers *k* novel interactions, is proportional to *k^−r^*, where *r* = 1.79 and 1.84 for PPIs and GIs, respectively, indicating that the per-study productivity roughly follows a power-law distribution (Figure 3 and Figure S1). We also observed that the number of co-authors per study increased over time (Figure 4), reflecting a general trend of increased collaboration in scientific research [10],[11]. Increase of productivity per author over time is not significant for PPIs, but significant for GIs (Figure S2). However, within virtually every year, per-author productivity is strongly negatively correlated with the number of co-authors of the study (Figure 5A and Table S1), suggesting that small research teams are more efficient than large teams at all times. Considering the possibility that researchers of small teams may publish fewer papers than those of large teams, we calculated accumulated productivity per-author in a five-year window. Again, authors of small teams consistently outperform those of large teams (Table S2) and this result remains qualitatively unchanged even when we consider the accumulated productivity of only those researchers who served at least once as the last author of a study in a five-year window (Table S3). However, the negative correlation between the productivity of a researcher and his/her mean team size appears to be weakening over the years (Figure 5B and Tables S1, S2 and S3).

### Important Subjects Were Studied Earlier

**Figure 6:**
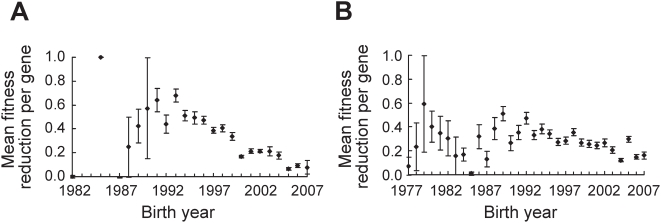
Genes appearing earlier in the (A) PPI network and (B) GI networks are more important to yeast. Pearson's rank correlation coefficient between the birth year of a gene in a network and the fitness reduction upon gene deletion is −0.28 (*n* = 4553, two-tail *P* = 7.6×10^−81^) for the PPI network and 0.14 (*n* = 3542, two-tail *P* = 7.4×10^−17^) for the GI network.

The ∼6000 yeast genes have been individually deleted to examine their functional importance, which is defined by the amount of reduction in the fitness of yeast caused by each deletion [12]. We traced the first year of appearance (birth year) of each gene in the PPI and GI networks, and found that genes appearing earlier in the networks (old genes) are more important than those appearing later (young genes) (Figure 6). One possible explanation of this phenomenon is that a gene's importance arises from the sheer number of its interactions [13]–[15]; if each interaction has the same probability of discovery, highly interactive genes are incorporated into the knowledge network earlier simply because they have more interactions. However, we found that old genes are more important than young genes even when the number of now known interactions per gene is controlled for (Spearman's partial correlation coefficient ρ = 0.13, *P* = 1.8×10^−17^ for the PPI network; ρ = 0.10, *P* = 5.3×10^−9^ for the GI network; Table 1). This result remains unchanged when we further control for the level of gene expression (Table 1). Thus, important genes are studied earlier not simply because of their large numbers of interactions, but also because of their phenotypic importance that is beyond what is predicted from their numbers of interactions.

**Table 1:** Partial correlations among the birth year, degree, importance, and expression level of yeast genes.

	Relationships examined^a^	Spearman's correlation coefficient	*P*-value^b^
PPIs			
	birth year, degree | importance	−0.422	4.00E-196
	degree, importance | birth year	0.280	1.23E-82
	birth year, importance | degree	−0.126	1.75E-17
	birth year, importance | degree, expression level	−0.153	5.78E-24
GIs			
	birth year, degree | importance	−0.379	3.55E-123
	degree, importance | birth year	0.083	8.20E-07
	birth year, importance | degree	−0.098	5.34E-09
	birth year, importance | degree, expression level	−0.086	6.15E-07

a Birth year is the year during which the gene was first included into the PPI (or GI) network. Degree is the number of interactions the gene has in the PPI (or GI) network in year-2007. Importance is the amount of fitness reduction caused by the deletion of the gene in yeast. Expression level is the expression level of the gene in the mid-log phase of yeast growth measured by microarray. Relationship between two properties (shown before I) is studied when another one or two properties (shown after I) are controlled for.

b Two-tail test.

### Familiar Subjects Were Preferentially Studied

**Figure 7:**
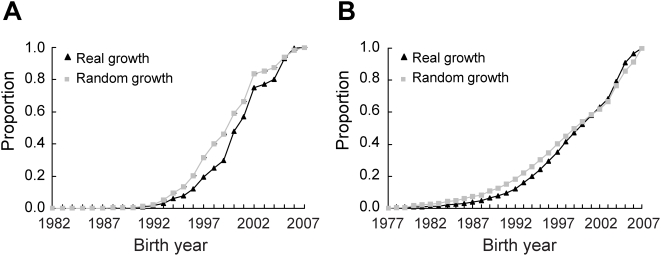
Reduced rates of discovery of new genes in the real growths of (A) the PPI network and (B) GI network, compared to the random growths. Shown on the Y-axis is the proportion of genes in the year-2007 network that were present in an earlier year. For the simulated random growth, the mean of 1000 replications is presented; the standard error is too small to see for all data points.

During the growth of the yeast biological knowledge network, a new interaction can introduce zero, one, or two genes into the network. Generally speaking, follow-up studies tend to discover interactions involving “pre-existing” genes while novel studies tend to discover interactions between previously “uncatalogued” genes [16]. We separately simulated the growths of yeast PPI and GI networks by randomizing the birth years of all interactions while conserving the number of new interactions discovered each year. Interestingly, the growth of gene number in the real networks lags behind the random expectation for many years (Figure 7), suggesting that, compared with the random process, actual researchers tend to focus on finding properties of known genes rather than those of new genes. We conducted 1000 simulations of random growth and found that the number of genes is 655.1±10 at 1995, the mid-point of PPI network growth, and this number is 676.1±14.6 for GI network at its mid-point of growth. Both numbers are significantly (*P*<0.001) larger than the observed numbers (390 for PPI network and 454 for GI network) in real growth. We also observed that the real growth pattern relative to the random pattern was reversed in recent years. However, this reserve is due to the fixation of total numbers of genes and interactions at year-2007 and does not suggest that the tendency of “novelty-aversion” has been reversed in research. The “novelty-aversion” phenomenon may arise from a high cost of novelty-seeking research and/or a high reward (or desire) for studying previously discovered genes [17]. As a consequence, the cohesiveness of the actual knowledge network is higher than that of a randomly growing network during the early years of yeast research (Figure S3).

### Heterogeneous and Episodic Growth of Knowledge Modules

**Figure 8:**
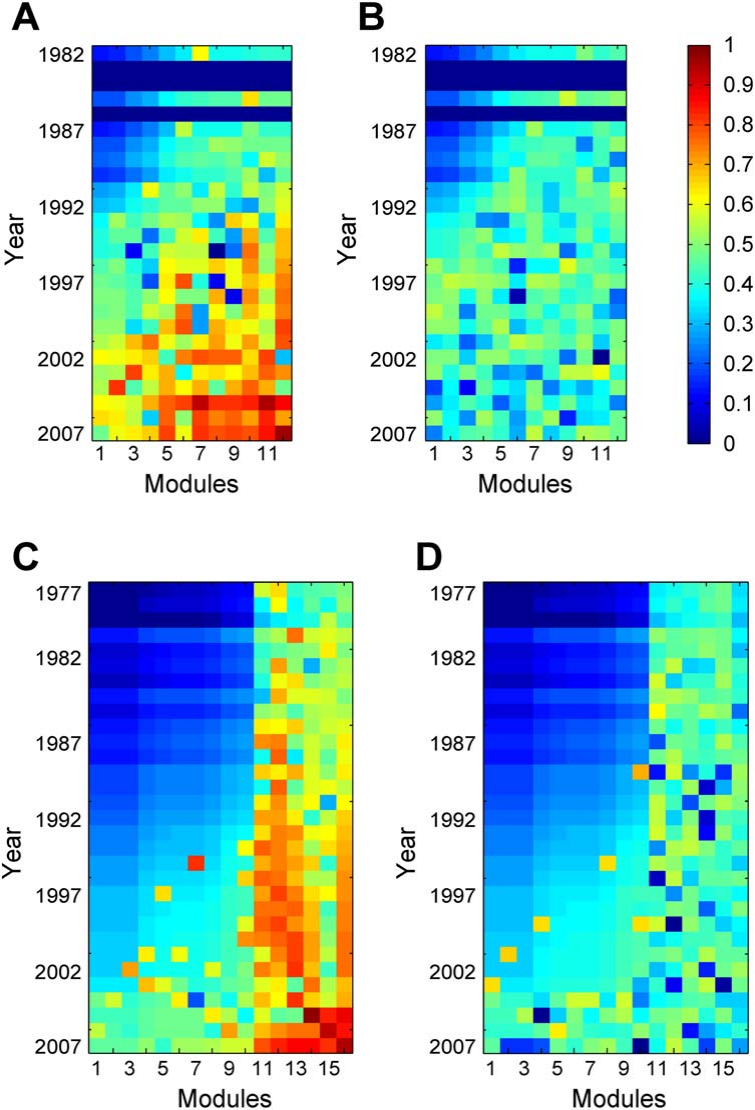
Greater deviations from homogenous module growths in the real (A) PPI and (C) GI networks than in the simulated randomly grown (B) PPI and (D) GI networks. Colors depict a transformed chi-squares value, 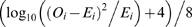, where *O_i_* is the observed growth of module *i* in a given year and *E_i_* is the expected (homogenous) growth given the total growth of the network in the year and the relative size of module *i* in year-2007. Reddish colors show greater deviations from homogenous growth, whereas bluish colors show smaller deviations.

**Figure 9:**
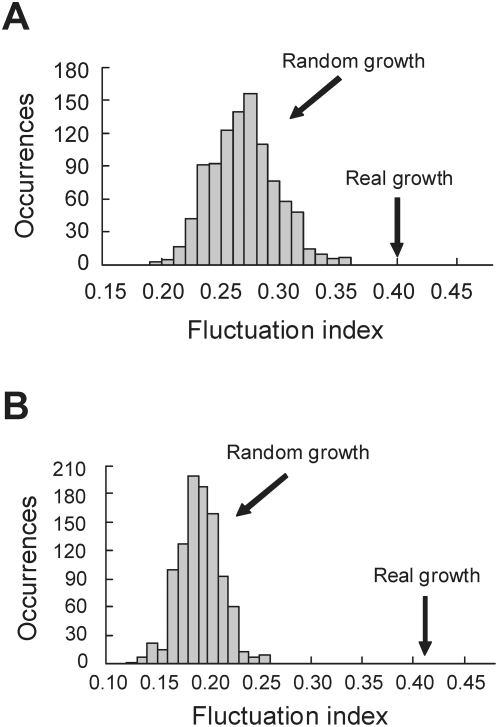
Significantly greater fluctuations of relative expansions of modules in (A) PPI and (B) GI networks than expected by chance. The chance expectation is illustrated by 1000 simulated random growths.

**Figure 10:**
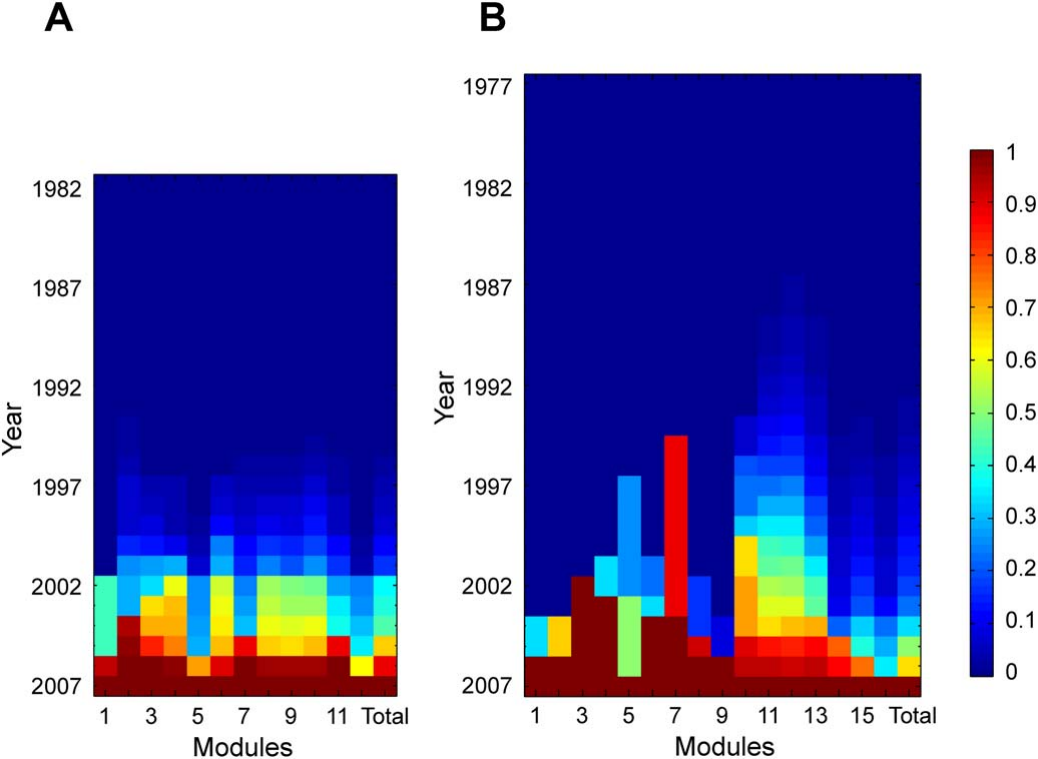
Different maturation status of different modules during the growths of the (A) PPI and (B) GI networks. The last column designated as “Total” in each panel shows the maturation status of the entire network. Color shows the maturation status, or completeness, of the growth of each module. All modules completed their growth at 2007, and thus are 100% completed in the bottom row.

Many complex networks are naturally divided into communities or modules, such that interactions within modules are much denser than those between modules [18]. The temporal PPI and GI data allow us to study the relative growths of different modules in a knowledge network compared to random growths. We identified 12 and 16 modules from the present-day PPI and GI networks, respectively [15] (see Materials and Methods). We transformed the network growth information into module growths by assigning one unit for every involved gene of a new interaction to the module that the gene belongs to. We then measured the deviation of the growth of each module from its expectation under homogenous growth, for each temporal PPI or GI network. Interestingly, although the network growth was contributed simultaneously by multiple modules in many years, the among-module heterogeneity in growth is striking, compared to random growths (Figure 8). For example, 4.7% of the PPI network growth was contributed by module #12 in year-2000, but this number becomes 70.8% in year-2007. The fluctuation index measured by mean Euclidean distance (see Materials and Methods) among these distributions is 0.40 and 0.42 for PPI and GI networks, respectively. Both are significantly larger than the expectations from simulated random growths of PPI (0.26±0.03) and GI (0.18±0.02) networks (*P*<0.001; Figure 9). This heterogeneous and episodic growth also leads to among-module variation in the maturation process of modules (Figure 10).

One wonders whether the observed heterogeneous and episodic growth of PPI and GI modules is owing to some recent large-scale studies that focused on genes involved in specific cellular functions; PPIs and GIs discovered from such studies are expected to be localized to certain knowledge modules rather than evenly distributed among all modules. To examine the effect of large-scale studies, we separately examined the network growth before and after year-1999. In the pre-1999 years, there was only 1 paper reporting >50 PPIs and 8 papers each reporting 20–50 PPIs, among the 919 papers on PPIs. Similarly, in this period, there were only 5 papers each reporting 20–50 GIs, among 1633 papers on GIs. In the post-1999 years, there were many large-scale studies. However, heterogeneous episodic growth of modules is found in both periods (Table S4). Thus, our observation is not simply a result of recent large-scale studies of specific cellular functions.

### Rapid Turnover of Knowledge Hubs

**Figure 11:**
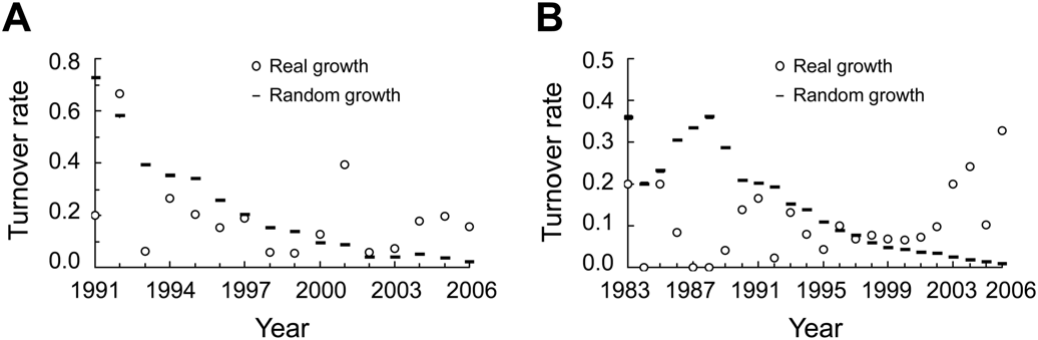
Constitutively high rate of turnover of temporal hubs during real network growth, compared with the decreasing rate of turnover during random network growth for (A) the PPI network and (B) GI network. For random growths, the mean of 1000 simulation replications is presented, and the error bar, which is almost invisible, shows one standard error.

**Figure 12:**
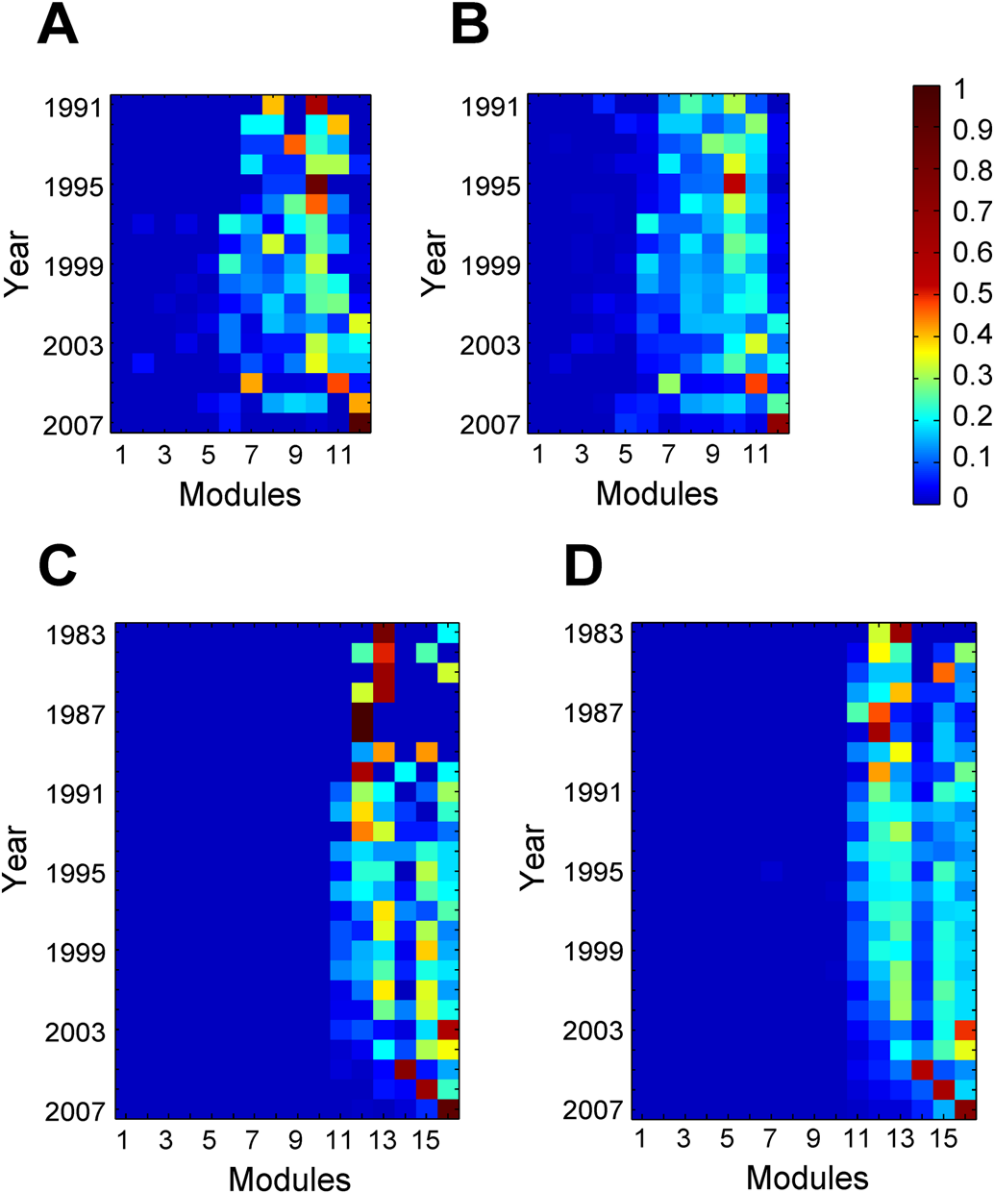
The birth of temporal hubs coincides with the pattern of modular expansion. (A) Among-module distribution of every year's new temporal hubs in the PPI network. (B) Among-module distribution of every year's new PPIs. (C) Among-module distribution of every year's new temporal hubs in the GI network. (D) Among-module distribution of every year's new GIs.

The heterogeneous and episodic growth of knowledge modules has an important consequence. Like many complex networks [19], connectivity is highly variable among nodes in the yeast PPI and GI networks. Most genes have one or a few interactions while a small fraction of genes have a very large number of interactions (Figure S4). Highly connected nodes (hubs) are known to be of both structural and functional importance to a network [13],[14],[19] (see also Table 1). Therefore, recognizing true hubs earlier would speed up the study of the network structure and function. However, hubs in today's network may not be hubs in the previous year's network and it is important to examine how stable hubs are during network growth. We arbitrarily define hubs in a given year as genes whose total connectivities in a network are among the top 10% of all available genes within the network at that time (only temporal networks with at least 50 genes are considered). We examined hub turnover in each year by computing the proportion of temporal hubs that become non-hubs in the following year. For both the PPI and GI networks, hub turnover rates are usually high (Figure 11). Surprisingly, hub stability did not increase with the growth of the network. For example, 32.5% of year-2006 GI hubs became non-hubs in 2007, and the corresponding number was 15.5% for year-2006 PPI hubs. This suggests that under the current mode of knowledge growth, it is difficult to predict true hubs before completion of network growth. By contrast, in the simulated random network growth, there is a trend of reduction in hub turnover over time. For example, in the GI network the turnover rate became <10% after year-1997 and <1% between year-2006 and 2007. The birth of temporal hubs appears to be strongly associated with heterogeneous expansions of modules (Figure 12).

The heterogeneous and episodic growth of network modules, and the related rapid hub turnover, are likely caused by a high reward (e.g., high-profile publications or large grants) for or biased interest in studying certain topics at certain times. For example, when a human disease-associated gene is identified, its yeast ortholog could be subject to intense studies immediately. Human syntaxin 8 was cloned in 1999 [20] and characterized as a member of the t-SNARE (target soluble N-ethylmaleimide sensitive factor attachment protein receptor) superfamily involved in vesicular trafficking and docking, a critical cellular process implicated in many human diseases [21]–[23]. Soon after the discovery, its yeast ortholog YAL014C was investigated and its 5 PPIs were identified by two studies in 2000 [24] and 2002 [25], respectively.

**Figure 13:**
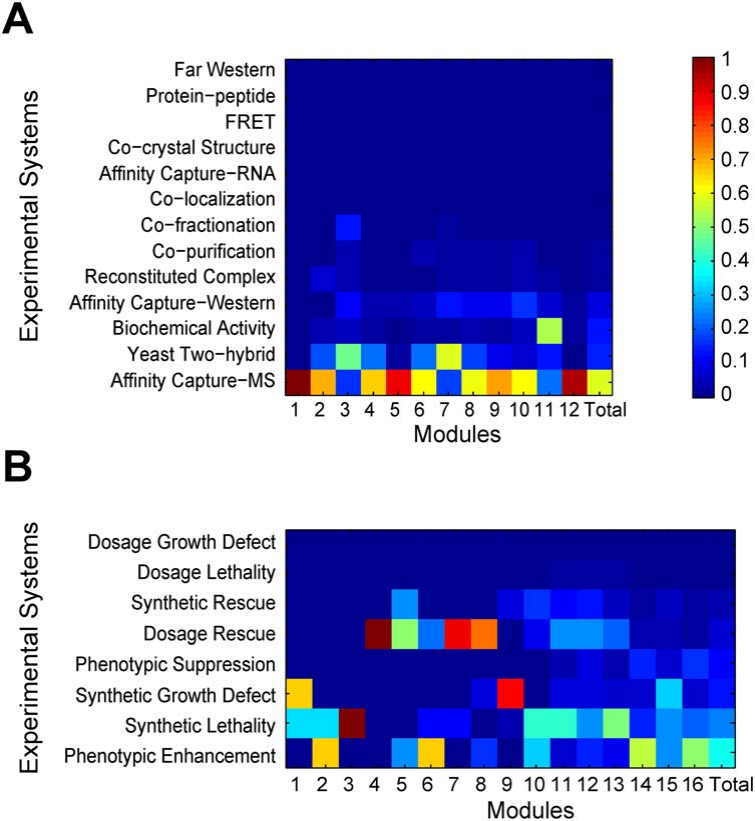
Interactions identified through different experimental systems are unevenly distributed among modules of the (A) PPI and (B) GI networks. The last column designated as “Total” in each panel shows the relative contribution of different experimental systems to the whole network. Note that since only novel interactions are considered and there is usually only one method in each publication, there is no novel interaction that was revealed by two methods in our analysis. Each module can be represented by a “method” vector, with each component of the vector being the fraction of interactions in the module that are discovered by each method. To examine how nonrandom different methods are in discovering interactions in different modules, we simulated the scenario in which all network modules are equally amenable to an experimental method, by randomizing the relationship between an interaction and the method used for its discovery. We calculated the total Euclidean distance between the method vectors of all pairs of modules. We conducted 1000 simulations for both PPI and GI networks, and the obtained Euclidean distances are 3.45±0.63 and 52.9±5.15, respectively. These distances are significantly (*P*<0.001) smaller than the observed distances in real networks (29.6 for PPI and 87.4 for GI).

In addition, different parts of a knowledge network are more likely to be discovered by different technologies that are invented at different times (Figure 13). For instance, in discovering PPIs, affinity approaches [26] tend to identify stable protein complexes while yeast two-hybrid assays [27] find dynamic interactions well. To further demonstrate this point, we directly compared two genome-wide studies that used either yeast two-hybrid assays [28] or affinity approaches [29] to discover PPIs. The across-module PPI distributions of the two studies are significantly different (Table S5). These results illustrate the importance of employing diverse approaches in knowledge exploration.

## Discussion

Although the PPI and GI networks analyzed here are still growing, they have been studied for ∼30 years and have encompassed most yeast genes. Thus, they serve as relatively good representations of the true and complete networks. For example, it is believed that we have already discovered ∼50% of all yeast PPIs [30]. Nevertheless, it is possible that we may have omitted some discoveries, although the BioGRID database, from which our data are acquired, is based on extensive literature searches [31]. To evaluate the potential effect of such omissions, we randomly excluded 10% of studies and repeated our analyses, and found that all major conclusions hold (data not shown). It should also be pointed out that, although the unbiased random network growth was based on the year-2007 networks, all principles should be applicable to the final true and complete networks.

The exponential growth shown in Figure 1 and the assumption that ∼50% of all PPIs in yeast have been identified predict that almost all yeast PPIs will have been discovered by year-2009, if the fraction of false positive discoveries does not increase with the rate of discovery. However, it is fully expected that both the current and future PPI and GI networks contain false interactions. Because false understanding exists in any type of knowledge, it will be interesting to study how false interactions affect the discoveries of true interactions. Unfortunately, BioGRID contains no information about previously reported interactions that are later dismissed. In fact, it is extremely difficult to falsify a previously reported interaction, because (i) the falsification requires one to test an interaction with exactly the same technique and condition as used in the initial experiment that discovered the interaction, and (ii) such falsification is by definition negative evidence for the existence of the interaction and therefore could be subject to other interpretations. Thus, at present it is difficult to evaluate how false interactions affect the growth of yeast biology.

In this work, we considered only the knowledge of the presence of an interaction and ignored detailed knowledge such as the strength of the interaction, the conditions under which the interaction occurs, and the biochemical or genetic basis of the interaction. It is difficult to analyze these types of knowledge at present because their structures are unclear. Paradigm shifts have been emphasized as an important mode of knowledge growth [2]. In the history of yeast research, the publication of the yeast genome sequence in 1996 [8] is widely thought to have triggered a paradigm shift from gene-based studies to genomic studies. However, such a shift in research scale and approach did not cause apparent changes in either the speed or pattern of discovery of new PPIs and GIs. Further analysis may reveal subtle signals of the paradigm shift that escaped our gross analysis. After all, our work represents just one step towards quantitative understanding of the tempo and mode of knowledge growth in the framework of network theories. Although the generality of our findings requires further evaluation, the lessons learned from this case study may help develop strategies for efficient knowledge exploration in the future.

## Materials and Methods

### Data

Yeast protein-protein interaction data and genetic interaction data were downloaded from BioGRID (http://www.thebiogrid.org). The publication year and author information for each interaction were extracted from NCBI (http://www.ncbi.nlm.nih.gov) using the PUBMED ID provided by BioGRID. Because we are interested in discoveries of new interactions, interactions that were reported in previous years were excluded. When a new interaction is reported by two or more publications of the same year, one of these publications was randomly chosen for further analyses. We measured the importance of a gene by the reduction in fitness of the yeast strain (i.e., growth rate) in rich medium (YPD) when the gene is deleted. The fitness data were downloaded from http://www-deletion.stanford.edu/YDPM/YDPM_index.html. The expression levels of yeast genes are measured at mid-log phase of growth and obtained from a previous study [32]. Authors with identical names were not differentiated. Although this practice necessarily introduced errors, it should not affect our results, because authors with common names and rare names are not expected to behave differently in research (e.g., they should participate in large teams with equal probabilities).

### Computational Analysis

Random network growth was simulated by randomizing the birth year of each interaction while keeping the number of newly discovered interactions unchanged for each year. Network modules were identified using simulated annealing, which has been shown to perform better than other module-separating algorithms [15]. The parameters used were: iteration factor = 0.1, cooling factor = 0.9, and final temperature = 10^−20^. For the PPI network, the giant component contains 99.72% of all genes and 99.98% of all interactions. The corresponding numbers are 98.18% and 99.89%, respectively, for the GI network. Relative growths of all modules in each year form a vector. The Euclidean distance between vectors of two consecutive years is then computed. The fluctuation index of a network is defined as the mean of Euclidean distances of all consecutive years. We transformed the network growth information into module growths by assigning one unit for every involved gene of a new interaction to the module that the gene belongs to. To measure the deviation of the actual growth of a module in a given year from the expected homogenous growth, we calculated a transformed chi-squares value, 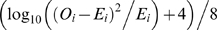, where *O_i_* is the observed growth of module *i* in a given year and *E_i_* is the expected (homogenous) growth given the total growth of the network in the year and the relative size of module *i* in year-2007. 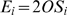, where *O* is the total number of interactions discovered in a given year and *S_i_* is the relative size measured by the sum of node degrees of module *i* to the entire network in year-2007. In short, for each year, the deviations from homogenous growth were calculated across modules.

## Supporting Information

Figure S1 Cumulative frequency distributions of productivity per study for (A) PPIs and (B) GIs.
(0.07 MB PDF)

Figure S2 Per-author productivity shows insignificant increase over time for publications reporting (A) PPIs but significant increase for publications reporting (B) GIs.
(0.19 MB PDF)

Figure S3 Cohesiveness of the (A) PPI and (B) GI networks is higher than expected under the random growth model during the early years of network growth.
(0.15 MB PDF)

Figure S4 The degree distribution of the (A) PPI and (B) GI networks.
(0.36 MB PDF)

Table S1 Small teams are more efficient than large teams in discovering new interactions.
(0.01 MB PDF)

Table S2 Researchers participating in larger teams have fewer discoveries of new interactions.
(0.01 MB PDF)

Table S3 Last authors of larger teams have fewer per-author discoveries of new interactions.
(0.01 MB PDF)

Table S4 Heterogeneous episodic growth of modules before and after year 1999
(0.01 MB PDF)

Table S5 Different methods differentially identify PPIs of different modules
(0.01 MB PDF)
