# Supplementary figures and images for: On the Growth of Scientific Knowledge: Yeast Biology as a Case Study
Source: PLoS Comput Biol. 2009 Mar 20;5(3):e1000320. doi: 10.1371/journal.pcbi.1000320 (PMC2649443; doi:10.1371/journal.pcbi.1000320)

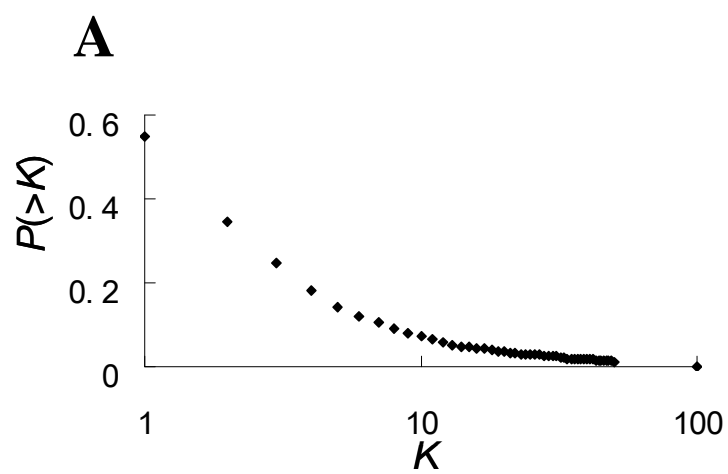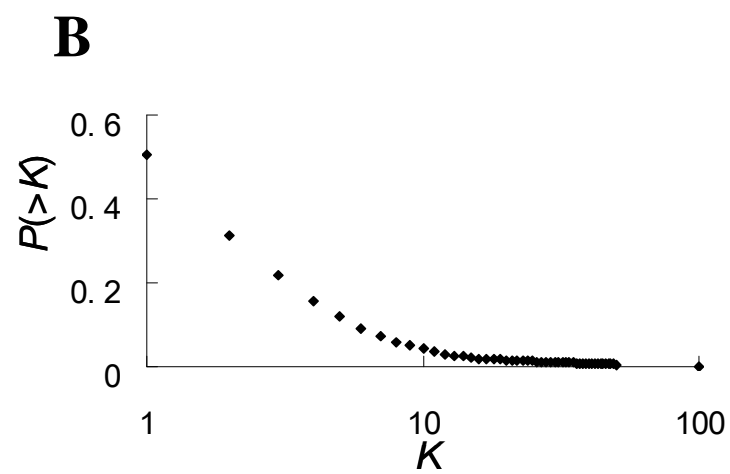

Fig. S1

Supplement: Figure S1 — Cumulative frequency distributions of productivity per study for (A) PPIs and (B) GIs. (0.07 MB PDF) [file pcbi.1000320.s001.pdf]

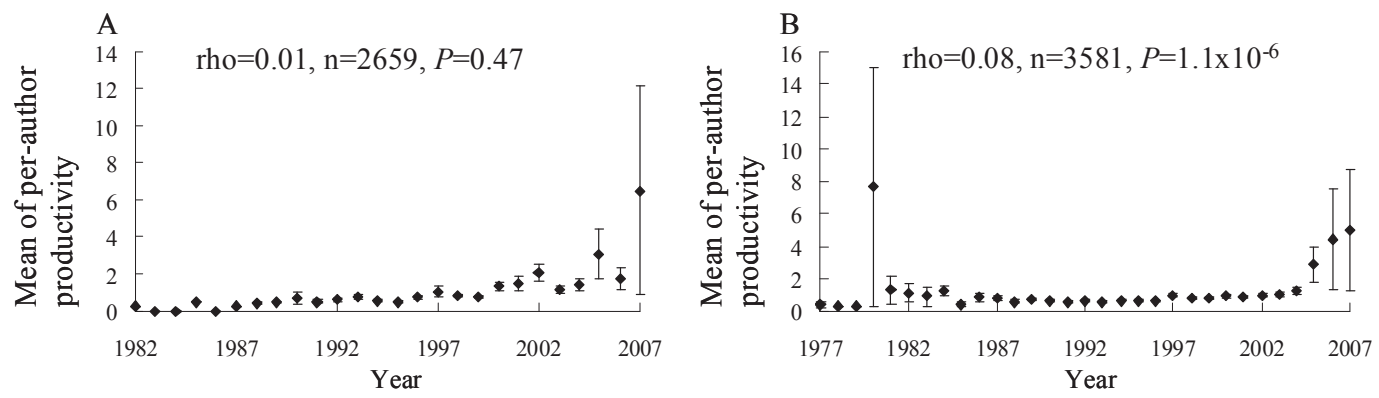

Fig. S2

Supplement: Figure S2 — Per-author productivity shows insignificant increase over time for publications reporting (A) PPIs but significant increase for publications reporting (B) GIs. (0.19 MB PDF) [file pcbi.1000320.s002.pdf]

**A**

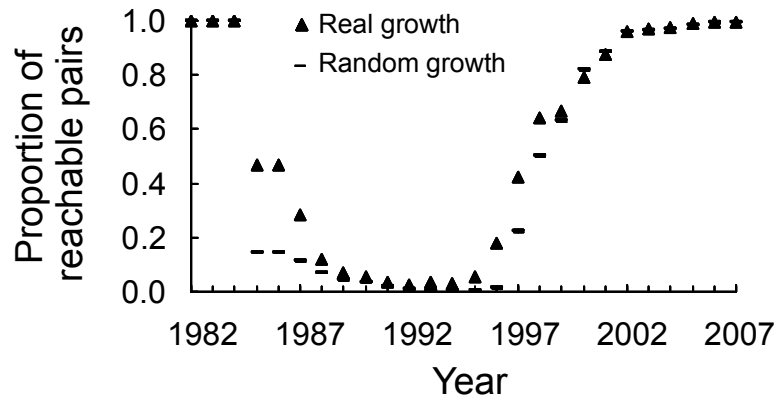

**B**

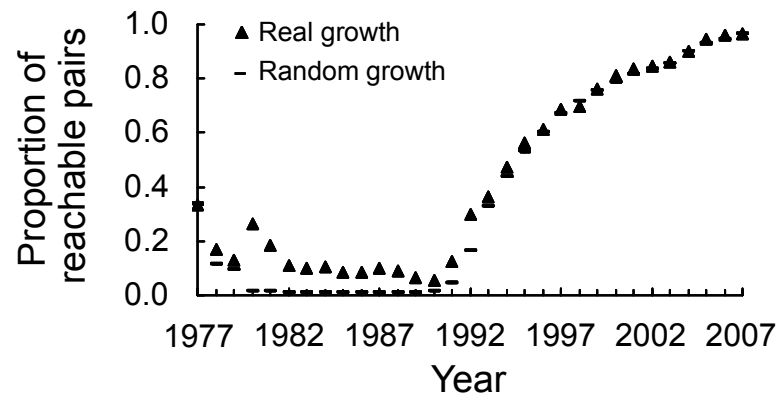

Fig. S3

Supplement: Figure S3 — Cohesiveness of the (A) PPI and (B) GI networks is higher than expected under the random growth model during the early years of network growth. (0.15 MB PDF) [file pcbi.1000320.s003.pdf]

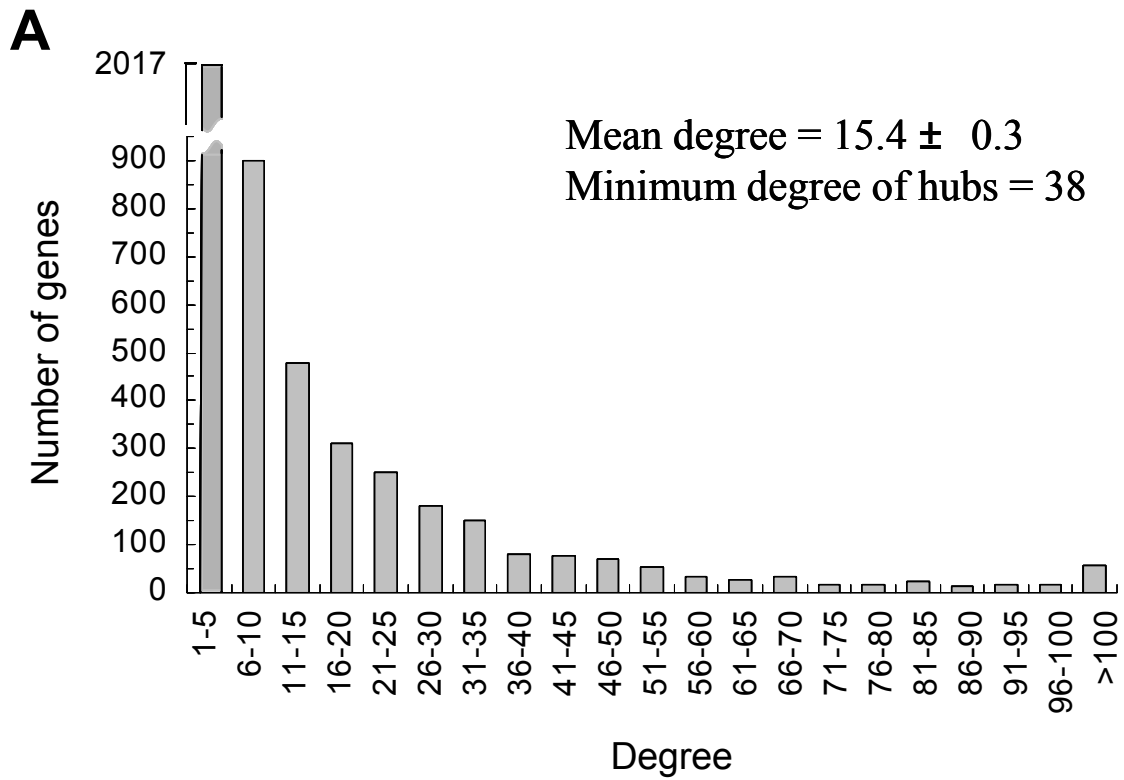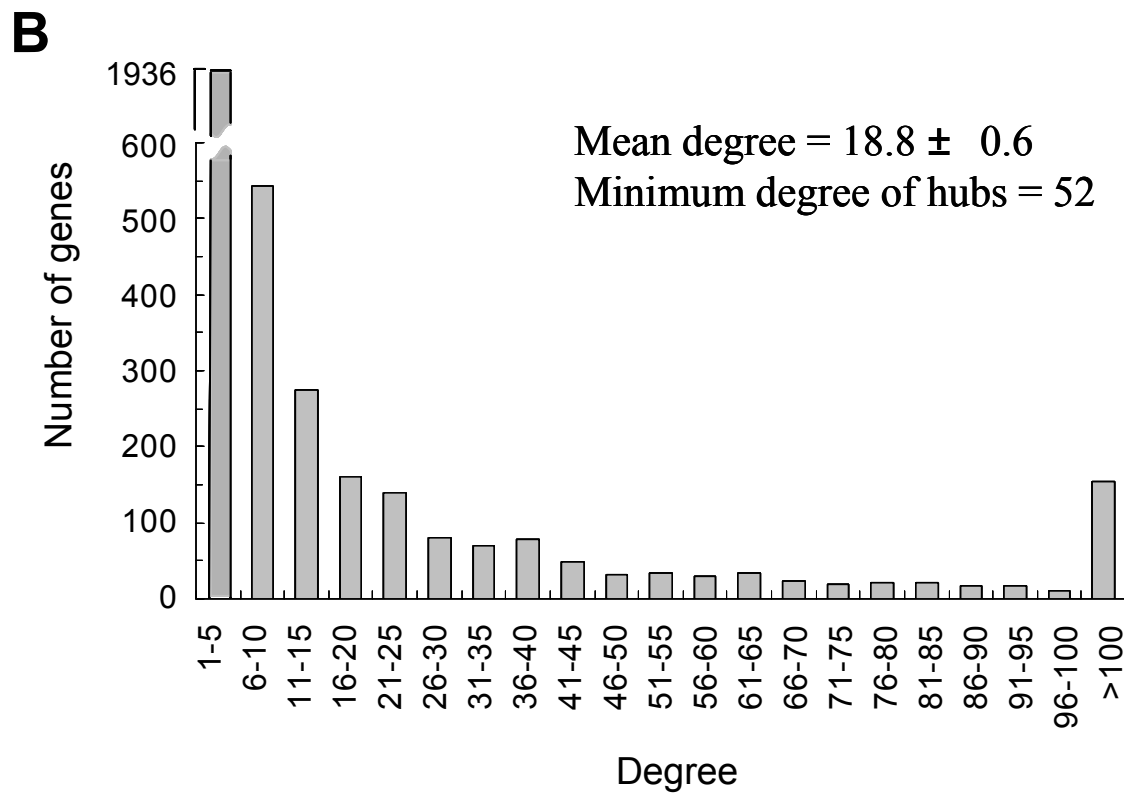

Fig. S4

Supplement: Figure S4 — The degree distribution of the (A) PPI and (B) GI networks. (0.36 MB PDF) [file pcbi.1000320.s004.pdf]
