# Supplementary material for: On the Growth of Scientific Knowledge: Yeast Biology as a Case Study
Source: PLoS Comput Biol. 2009 Mar 20;5(3):e1000320. doi: 10.1371/journal.pcbi.1000320 (PMC2649443; doi:10.1371/journal.pcbi.1000320)
Supplement: Table S1 — Small teams are more efficient than large teams in discovering new interactions. (0.01 MB PDF) [file pcbi.1000320.s005.pdf]

Table S1. Small teams are more efficient than large teams in discovering new interactions.

	Protein-protein interactions			Genetic interactions		
Year	# of public.	rho[a]	*P*-value	# of public.	rho[a]	*P*-value
1977	0			2	-1.000	1.00E+00
1978	0			3	-1.000	6.67E-01
1979	0			1		
1980	0			2	-1.000	1.00E+00
1981	0			3	-0.866	6.67E-01
1982	1			6	-0.955	1.67E-02
1983	0			3	-1.000	6.67E-01
1984	0			10	-0.469	1.72E-01
1985	1			8	-0.704	5.95E-02
1986	0			12	-0.580	4.79E-02
1987	1			17	-0.441	7.66E-02
1988	3	-1.000	6.67E-01	19	-0.788	6.20E-05
1989	5	-1.000	4.00E-01	35	-0.335	4.93E-02
1990	2	-1.000	1.00E+00	39	-0.490	1.54E-03
1991	11	-0.664	2.60E-02	53	-0.563	1.16E-05
1992	17	-0.543	2.44E-02	68	-0.714	7.93E-12
1993	34	-0.642	4.23E-05	110	-0.667	2.00E-15
1994	68	-0.438	1.89E-04	118	-0.622	5.30E-14
1995	73	-0.514	3.34E-06	158	-0.636	<1.00E-15
1996	116	-0.602	8.54E-13	201	-0.602	<1.00E-15
1997	179	-0.467	4.53E-11	216	-0.607	<1.00E-15
1998	210	-0.424	1.36E-10	273	-0.634	<1.00E-15
1999	198	-0.440	9.01E-11	276	-0.566	<1.00E-15
2000	252	-0.477	1.00E-15	298	-0.531	<1.00E-15
2001	244	-0.375	1.39E-09	256	-0.486	<1.00E-15
2002	240	-0.305	1.42E-06	297	-0.496	<1.00E-15
2003	231	-0.378	2.89E-09	252	-0.475	1.00E-15
2004	226	-0.292	8.09E-06	280	-0.425	1.09E-13
2005	242	-0.173	6.88E-03	246	-0.340	4.62E-08
2006	215	-0.391	2.75E-09	204	-0.383	1.54E-08
2007	90	-0.355	6.08E-04	115	-0.499	1.40E-08

[a] Spearman's rank correlation coefficient between the number of new interactions discovered per author in a study and the number of co-authors of the study within each year that has at least two studies.
