# Supplementary material for: On the Growth of Scientific Knowledge: Yeast Biology as a Case Study
Source: PLoS Comput Biol. 2009 Mar 20;5(3):e1000320. doi: 10.1371/journal.pcbi.1000320 (PMC2649443; doi:10.1371/journal.pcbi.1000320)
Supplement: Table S2 — Researchers participating in larger teams have fewer discoveries of new interactions. (0.01 MB PDF) [file pcbi.1000320.s006.pdf]

Table S2. Researchers participating in larger teams have fewer discoveries of new interactions.

	Protein-protein interactions			Genetic interactions		
Year	# of authors	rho[a]	*P*-value	# of authors	rho[a]	*P*-value
1977-1981				28	-0.913	1.35E-11
1982-1986	10	NA[b]	NA[b]	100	-0.571	5.57E-10
1987-1991	62	-0.686	7.32E-10	387	-0.396	1.00E-15
1992-1996	967	-0.465	<1.00E-15	1773	-0.496	<1.00E-15
1997-2001	3128	-0.224	<1.00E-15	3562	-0.435	<1.00E-15
2002-2006	3926	-0.039	1.38E-02	4140	-0.291	<1.00E-15

[a] Spearman's rank correlation coefficient between the average number of coauthors on the publications of an author within a five year window and the total number of new interactions discovered by the author within the same five years. We assume that each author discovers $1/n$ fraction of interactions reported in a paper with n coauthors. *P*-values are from two-tail tests.

[b] Not applicable because only two publications existed in this period of time.
