# Supplementary material for: On the Growth of Scientific Knowledge: Yeast Biology as a Case Study
Source: PLoS Comput Biol. 2009 Mar 20;5(3):e1000320. doi: 10.1371/journal.pcbi.1000320 (PMC2649443; doi:10.1371/journal.pcbi.1000320)
Supplement: Table S3 — Last authors of larger teams have fewer per-author discoveries of new interactions. (0.01 MB PDF) [file pcbi.1000320.s007.pdf]

Table S3. Last authors of larger teams have fewer per-author discoveries of new interactions.

	Protein-protein interactions			Genetic interactions		
Year	# of last authors	rho[a]	*P*-value	# of last authors	rho[a]	*P*-value
1977-1981				11	-0.721	1.22E-02
1982-1986	2	NA[b]	NA[b]	33	-0.358	4.10E-02
1987-1991	20	-0.476	3.37E-02	103	-0.086	3.88E-01
1992-1996	212	-0.294	1.40E-05	359	-0.280	6.73E-08
1997-2001	578	-0.047	2.56E-01	715	-0.250	1.28E-11
2002-2006	644	0.054	1.71E-01	737	-0.129	4.67E-04

[a] Spearman's rank correlation coefficient between the average number of coauthors on the publications of an author within a five year window and the total number of new interactions discovered by the author within the same five years. We assume that each author discovers $1/n$ fraction of interactions reported in a paper with n coauthors. Here we analyze only those who are the last author of at least one publication in a 5-year window. *P*-values are from two-tail tests.

[b] Not applicable because only two publications existed in this period of time.
