# Supplementary material for: On the Growth of Scientific Knowledge: Yeast Biology as a Case Study
Source: PLoS Comput Biol. 2009 Mar 20;5(3):e1000320. doi: 10.1371/journal.pcbi.1000320 (PMC2649443; doi:10.1371/journal.pcbi.1000320)
Supplement: Table S4 — Heterogeneous episodic growth of modules before and after year 1999 (0.01 MB PDF) [file pcbi.1000320.s008.pdf]

Table S4. Heterogeneous episodic growth of modules before and after year 1999.

	Before 1999	From 1999
PPI network		
Fluctuation index (Observed)[a]	0.23	0.31
Fluctuation index (Simulated)[b]	0.11±0.01	0.023±0.003
P -value[c]	< 0.001	< 0.001
GI network		
Fluctuation index (Observed)[a]	0.27	0.34
Fluctuation index (Simulated)[b]	0.13±0.02	0.024±0.004
P -value[c]	< 0.001	< 0.001

[a] See Materials and Methods for the definition of fluctuation index.

[b] From simulated random growth.

[c] Determined from 1000 simulations.
