# Supplementary material for: On the Growth of Scientific Knowledge: Yeast Biology as a Case Study
Source: PLoS Comput Biol. 2009 Mar 20;5(3):e1000320. doi: 10.1371/journal.pcbi.1000320 (PMC2649443; doi:10.1371/journal.pcbi.1000320)
Supplement: Table S5 — Different methods differentially identify PPIs of different modules (0.01 MB PDF) [file pcbi.1000320.s009.pdf]

Table S5. Different methods differentially identify PPIs of different modules.

Number of PPIs discovered in each module	Y2H[a]	Affinity[b]
Module #1	23	0
Module #2	2	11
Module #3	6	25
Module #4	91	94
Module #5	439	12
Module #6	578	85
Module #7	444	95
Module #8	1751	183
Module #9	1123	163
Module #10	1933	127
Module #11	1235	293
Module #12	1613	34

Chi-square=894.4, degree of freedom=11, $P < 10^{-180}$.

[a] Data from reference [28].

[b] Data from reference [29].
